# Leaflet Morphology Is More Strongly Associated with Atrial Functional Mitral Regurgitation Severity than Annular Dilation: A Three-Dimensional Transesophageal Echocardiographic Study

**DOI:** 10.3390/diagnostics16142228

**Published:** 2026-07-16

**Authors:** Andrei-Alexandru Nour, Diana-Ruxandra Hădăreanu, Despina-Manuela Toader, Călin-Dinu Hădăreanu, Maria-Livia Iovănescu, Anca Mihu-Marinescu, Georgică-Costinel Târtea, Ionuț Donoiu, Oana Munteanu-Mirea, Petre-Alexandru Cojocaru, Marius-Bogdan Novac, Octavian Istrătoaie, Cristina Florescu

**Affiliations:** 1 Doctoral School, University of Medicine and Pharmacy of Craiova, 2 Petru Rares St., 200349 Craiova, Romania; 2 Department of Cardiology, Filantropia Clinical Hospital, 28 Sararilor St., 200516 Craiova, Romania; 3 Department of Cardiology, University of Medicine and Pharmacy of Craiova, 2 Petru Rares St., 200349 Craiova, Romania; 4 Department of Cardiology, Clinical Emergency County Hospital of Craiova, 1 Tabaci St., 200642 Craiova, Romania; 5 Department of Cardiovascular Surgery, Clinical Emergency County Hospital of Craiova, 1 Tabaci St., 200642 Craiova, Romania; 6 Department of Anesthesiology and Intensive Care, University of Medicine and Pharmacy of Craiova, 2 Petru Rares St., 200349 Craiova, Romania

**Keywords:** atrial fibrillation, atrial functional mitral regurgitation, three-dimensional echocardiography, transesophageal echocardiography, mitral valve, leaflet morphology

## Abstract

**Background**: Atrial functional mitral regurgitation (AFMR) results from left atrial (LA) remodeling and mitral annular dilation in patients with atrial fibrillation and preserved left ventricular function. While annular dilation is considered the primary mechanism, the role of leaflet morphology in determining regurgitation severity remains incompletely characterized. We hypothesized that leaflet morphology, rather than annular dilation alone, is more strongly correlated with AFMR severity. **Methods**: We prospectively studied 113 consecutive patients with persistent atrial fibrillation and AFMR who underwent comprehensive three-dimensional transesophageal echocardiography (3D TEE). Mitral valve geometry was analyzed using dedicated software (EchoPAC v.206, 3D MVQ Analysis). Patients were classified according to MR severity: non-significant (grade 0–1) versus moderate or severe (grade 2–3). Logistic regression identified predictors of moderate or severe AFMR. **Results**: Moderate or severe MR was present in 57 patients (50.4%). Compared with patients with non-significant MR, those with moderate or severe regurgitation exhibited larger annular dimensions (3D annular area 12.7 vs. 11.4 cm^2^, *p* = 0.005), reduced non-planar angle (145° vs. 149°, *p* = 0.027), greater leaflet areas, and increased tethering parameters. Anterior leaflet length was markedly greater in the moderate/severe group (2.7 vs. 2.4 cm, *p* = 0.001). In different multivariable analyses models adjusting for age, sex, LA diameter, and 3D annular area, anterior leaflet length (OR 3.16 per SD, 95% CI 1.65–6.61, *p* = 0.001), anterior leaflet area (OR 3.42 per SD, 95% CI 1.48–8.74, *p* = 0.006), and posterior leaflet length (OR 0.39 per SD, 95% CI 0.15–0.86, *p* = 0.043) remained independently associated with moderate or severe AFMR. ROC analysis demonstrated good discriminative ability for anterior leaflet length (AUC 0.746, with an optimal threshold of 2.55 cm, sensitivity 75%, specificity 68%), and anterior leaflet area (AUC 0.680, and an optimal cut-off value of 5.75 cm^2^, sensitivity 70.2%, specificity of 64.3%). **Conclusions**: In patients with AFMR, anterior leaflet dimensions assessed by 3D TEE are the strongest independent predictors of moderate or severe regurgitation, outperforming annular parameters. These measurements may represent practical tools for risk stratification and patient selection for intervention.

## 1. Introduction

Atrial functional mitral regurgitation (AFMR) has emerged as a distinct clinical entity characterized by mitral regurgitation (MR) occurring in the context of atrial fibrillation (AF) and left atrial (LA) enlargement, in the absence of primary leaflet pathology or significant left ventricular (LV) systolic dysfunction [1,2,3]. Unlike ventricular functional mitral regurgitation (VFMR), which results from LV dilation and papillary muscle displacement, AFMR is driven primarily by LA remodeling and consequent mitral annular dilation [1,2,3]. The clinical significance of AFMR has been increasingly recognized, with recent data demonstrating that patients with moderate or severe AFMR experience high rates of heart failure (HF) hospitalization and mortality [4,5].

The pathophysiology of AFMR has traditionally been attributed to mitral annular dilation secondary to LA enlargement [1,2,3]. The loss of atrial contractility in AF leads to chronic elevation of LA pressure, progressive atrial remodeling, and subsequent expansion of the mitral annulus, which is anatomically contiguous with the LA wall [3]. Additionally, flattening of the normal saddle-shaped annular configuration increases the projected annular area, further compromising leaflet coaptation [3].

However, annular dilation alone does not fully explain the heterogeneity in MR severity observed among patients with AF. Recent three-dimensional (3D) echocardiographic studies have demonstrated that mitral leaflet area increases in response to annular dilation as an adaptive mechanism to maintain coaptation. Kim et al. [6] showed that this adaptive leaflet growth plateaus at higher degrees of annular dilation, resulting in inadequate leaflet coverage and more severe regurgitation. Kagiyama et al. [7] further demonstrated that patients with significant AFMR have lower leaflet-to-annulus area ratios than those without significant regurgitation, establishing the paradigm that leaflet adaptation relative to annular size is a critical determinant of coaptation adequacy.

Despite these advances, prior studies have predominantly used 3D transthoracic echocardiography, which may be limited by suboptimal image quality in patients with AF and irregular R-R intervals. Furthermore, most investigations have focused on leaflet area rather than leaflet length, and the relative importance of different structural parameters in determining AFMR severity has not been systematically evaluated using multivariable analysis.

Three-dimensional transesophageal echocardiography (3D TEE) offers superior spatial resolution for precise quantification of mitral valve geometry, enabling accurate measurement of annular dimensions, leaflet parameters, and tethering indices. Multimodality imaging has similarly transformed the assessment of tricuspid valve anatomy and functional regurgitation, emphasizing the importance of advanced structural imaging in atrioventricular valve disease [8]. The transesophageal approach is particularly advantageous for leaflet length measurement, which requires clear delineation of the annular hinge point and leaflet free edge—structures that may be suboptimally visualized with transthoracic imaging [9,10].

We hypothesized that leaflet morphology, rather than annular dilation alone, is more strongly associated with AFMR severity, and that leaflet dimensions—including both length and area—may outperform annular parameters in predicting regurgitation severity. The aims of this study were: (1) to comprehensively characterize the 3D structural phenotype of moderate or severe AFMR using high-resolution 3D TEE; (2) to identify the strongest independent predictors of AFMR severity using multivariable analysis; and (3) to evaluate the discriminative performance of key structural parameters for identifying patients with clinically significant regurgitation.

## 2. Methods

### 2.1. Study Design and Population

This was a prospective, observational study conducted at the Clinical Emergency County Hospital of Craiova (Romania), designed to evaluate the structural and valvular changes induced by persistent AF, using advanced 3D echocardiography. Consecutive eligible patients with persistent AF undergoing clinically indicated TEE prior to electrical cardioversion were prospectively recorded in an institutional clinical imaging database. As part of standard hospital admission procedures, all patients signed written informed consent permitting anonymized use of their clinical and imaging data for research purposes. The REMO-FIB (“The assessment of cardiac remodeling by advanced imaging techniques in patients with atrial fibrillation”) project represented a subsequent focused research project designed to evaluate the structural predictors of AFMR in this population. For the present analysis, we selected the subgroup of patients meeting the following inclusion criteria: (1) presence of AFMR, defined according to the 2021 ESC/EACTS Guidelines for the Management of Valvular Heart Disease as functional MR occurring in the context of AF and LA dilation, with preserved or mildly reduced LV systolic function, and absence of primary leaflet abnormalities or significant LV dilation; and (2) availability of 3D TEE datasets of adequate quality for offline mitral valve analysis. Exclusion criteria were: (1) primary mitral valve disease (degenerative, rheumatic, or endocarditic); (2) prior mitral valve surgery or transcatheter intervention; (3) LV ejection fraction < 40%; (4) significant LV dilation (end-diastolic diameter > 65 mm); (5) regional wall motion abnormalities suggesting ischemic cardiomyopathy; (6) more than mild aortic valve disease; (7) congenital heart disease; and (8) inadequate echocardiographic image quality precluding 3D analysis. After applying these criteria, 113 patients were included in the final study population. The study was approved by the Ethics Committee of the University of Medicine and Pharmacy of Craiova, Romania (approval number 241, date of approval: 25 October 2023; additional project-specific approval number 117; date of approval: 10 February 2025), and conducted in accordance with the Declaration of Helsinki.

### 2.2. Clinical Data Collection

Baseline clinical data were collected prospectively at the time of enrollment, including demographics, AF characteristics (pattern, duration, EHRA symptom class), cardiovascular risk factors, comorbidities, and current medications. The CHA_2_DS_2_-VASc and HAS-BLED scores were calculated according to standard definitions. HF was classified according to the 2021 European Society of Cardiology Guidelines [11] based on LV ejection fraction. Laboratory data including creatinine, estimated glomerular filtration rate (CKD-EPI equation), and hemoglobin were recorded.

### 2.3. Standard Echocardiographic Assessment

All patients underwent clinically indicated TEE for exclusion of LA thrombi before electrical cardioversion, while in AF, using commercially available ultrasound systems (Vivid E95, GE Healthcare, Horten, Norway) equipped with matrix-array transesophageal transducers (6VT-D). Standard two-dimensional and Doppler echocardiographic measurements were performed according to current recommendations of the European Association of Cardiovascular Imaging [10]. MR severity was graded using an integrative multiparametric approach, according to current guidelines recommendations [10]. MR was categorized as grade 0 (none/trace), grade 1 (mild), grade 2 (moderate), or grade 3 (severe). For the purposes of this analysis, patients were dichotomized into two groups: non-significant MR (grade 0–1) and moderate or severe MR (grade 2–3). Tricuspid regurgitation (TR) was graded using a similar integrative approach, and significant TR was defined as grade ≥ 2.

### 2.4. Three-Dimensional Transesophageal Echocardiographic Acquisition

3D TEE was performed under conscious sedation with continuous monitoring of oxygen saturation, heart rate, and blood pressure. Dedicated 3D datasets of the mitral valve were acquired from the mid-esophageal position using electrocardiographically gated full-volume acquisitions. Care was taken to include the entire mitral valve apparatus within the pyramidal volume, with optimization of gain, compression, and frame rate settings to maximize image quality. Multiple acquisitions were obtained to ensure adequate image quality, and the dataset with the best visualization of the mitral valve was selected for offline analysis.

### 2.5. Three-Dimensional Mitral Valve Apparatus Analysis

**Figure 1:**
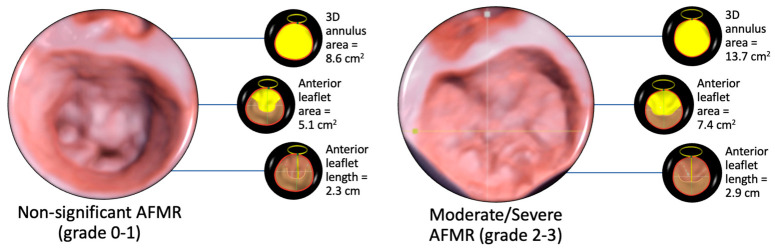
Representative examples of three-dimensional transesophageal echocardiographic mitral valve reconstruction in patients with non-significant AFMR (grade 0–1) and moderate/severe AFMR (grade 2–3). The figure illustrates larger annular dimensions and altered anterior leaflet morphology in clinically significant AFMR. Measurements were obtained using dedicated 3D mitral valve quantification software (EchoPAC v206, 3D MVQ Analysis). Abbreviations: 3D, three-dimensional; AFMR, atrial functional mitral regurgitation.

Offline analysis of 3D datasets was performed using dedicated software (EchoPAC version 206, 3D MVQ Analysis, GE Healthcare) by experienced operators blinded to clinical data. Representative examples of 3D mitral valve reconstruction and quantitative analysis are shown in Figure 1. The analysis was performed on mid-systolic frames to capture maximal leaflet coaptation. Contemporary multimodality imaging approaches increasingly emphasize dedicated 3D quantification and advanced display techniques for precise assessment of valvular geometry and coaptation mechanisms in functional regurgitation [12]. The 3D mitral valve quantification protocol included systematic assessment of annular geometry, leaflet morphology, coaptation geometry, and tethering parameters, as follows:

• Annular Parameters: 3D annular area, two-dimensional (projected) annular area, annular perimeter, anteroposterior diameter, posteromedial-anterolateral (commissural) diameter, inter-trigonal distance, annular height (the perpendicular distance between the highest and lowest points of the annulus), non-planar angle (the angle between the anterior and posterior portions of the annulus, reflecting saddle-shape configuration), sphericity index (ratio of anteroposterior to commissural diameter), mitral annular excursion, mitral annular maximal velocity, annulus area fraction (the percentage change in annular area during the cardiac cycle), and mitral-aortic angle.
• Leaflet Parameters: Anterior leaflet area, posterior leaflet area, anterior leaflet length (measured from the annular hinge point to the leaflet free edge at the A2 segment), posterior leaflet length (measured from the annular hinge point to the leaflet free edge at the P2 segment), anterior leaflet angle (the angle between the anterior leaflet and the annular plane), and posterior leaflet angle (the angle between the posterior leaflet and the annular plane).
• Coaptation Parameters: Anterior closure line length (the length of the coaptation zone along the anterior leaflet) and posterior closure line length (the length of the coaptation zone along the posterior leaflet).
• Tethering Parameters: Tenting height (the perpendicular distance from the annular plane to the coaptation point), tenting area (the area enclosed between the annular plane and the leaflets in the apical long-axis view), tenting volume (the three-dimensional volume enclosed between the annular plane and the leaflets), and tenting volume fraction (tenting volume as a percentage of total left ventricular volume).

### 2.6. Reproducibility Analysis

To assess the reproducibility of anterior leaflet length, and area, and 3D annulus area measurements, intra-observer and inter-observer variability were evaluated in a randomly selected subset of 20 patients. For intra-observer variability, the same operator (D.-R.H.) repeated the measurements at least 2 weeks apart, blinded to the initial results. For inter-observer variability, a second experienced operator (A.M.-M.) independently analyzed the same datasets, blinded to the first operator’s measurements. Reproducibility was assessed using intraclass correlation coefficients (ICC), and Bland–Altman analysis.

### 2.7. Statistical Analysis

Continuous variables were expressed as median and interquartile range (IQR), given the non-normal distribution of most parameters assessed by Shapiro–Wilk test. Categorical variables were reported as counts and percentages. Comparisons between groups (non-significant MR vs. moderate or severe MR) were performed using the Mann–Whitney U test for continuous variables and the chi-square test or Fisher’s exact test, as appropriate, for categorical variables. Univariable logistic regression analysis was used to identify clinical and echocardiographic predictors of moderate or severe MR. Continuous predictors entered into logistic regression were standardized per one standard deviation increase to allow comparison of effect sizes across variables with different units and scales. Results were expressed as odds ratios (OR) with 95% confidence intervals (CI). Exploratory multivariable logistic regression models were constructed to identify independent predictors of moderate or severe MR. Variable selection was based on clinical plausibility, mechanistic relevance, and univariable significance, while avoiding inclusion of highly collinear variables. A basal model (Model 1) was first constructed including age, sex, LA diameter, and 3D annular area. To assess the independent contribution of leaflet geometry beyond annular remodeling, each leaflet parameter (anterior leaflet area, anterior leaflet length, posterior leaflet area, and posterior leaflet length) was then added separately to this basal model (Models 2–5). Receiver operating characteristic (ROC) analysis was performed to assess the discriminative performance of anterior leaflet length and area for identifying moderate or severe MR. The area under the curve (AUC) with 95% confidence interval was calculated. The optimal threshold was determined using the Youden index (sensitivity + specificity − 1). To assess model robustness, multicollinearity was evaluated using variance inflation factors (VIFs). Model calibration was assessed using the Hosmer–Lemeshow goodness-of-fit test and the Brier score. Given the exploratory nature of the study, an additional sensitivity analysis including all leaflet geometry parameters simultaneously was performed to evaluate their independent contribution to AFMR severity. A two-sided *p*-value < 0.05 was considered statistically significant. All statistical analyses were performed using R version 4.5.3. No formal sample size calculation was performed because this was an exploratory study including all consecutive eligible patients during the study period.

## 3. Results

### 3.1. Study Population

A total of 113 patients with AFMR who underwent 3D TEE were included in the present analysis. The baseline characteristics of the study population are summarized in Table 1. The median age was 68 years (interquartile range 63–73), and 38.1% were female. Comorbidities were prevalent, including hypertension (89.3%), dyslipidemia (85.0%), diabetes mellitus (34.5%), and chronic kidney disease (34.5%). HF was present in 80.5% of patients. LV systolic function was generally preserved, with a median ejection fraction of 51% (50–55%), consistent with the atrial functional phenotype. Moderate or severe MR was present in 57 patients (50.4%), whereas the remaining 56 patients (49.6%) had no or mild regurgitation.

### 3.2. Clinical and Echocardiographic Characteristics According to MR Severity

When patients were stratified according to MR severity (Table 1), those with moderate or severe regurgitation were significantly older (median 70 vs. 67 years, *p* = 0.044) and exhibited larger LA dimensions (median 46 vs. 44 mm, *p* = 0.005) and right atrial dimensions (median 41 vs. 39 mm, *p* = 0.041). Patients with moderate or severe AFMR had significantly longer duration of AF compared with those with non-significant MR (*p* = 0.002). Patients with more severe MR also had lower hemoglobin levels (median 13.6 vs. 14.7 g/dL, *p* = 0.003). No significant differences were observed with respect to sex distribution, global comorbidity burden, or LV ejection fraction, supporting the concept that MR severity in this cohort was related to atrial and valvular remodeling rather than overt impairment of LV systolic function. Significant TR (grade ≥ 2) was present in 31.9% of the overall cohort and was significantly more prevalent among patients with moderate or severe MR (43.9% vs. 19.6%, *p* = 0.010), consistent with a broader biatrial remodeling phenotype affecting both atrioventricular valves. Regarding medical therapy, patients with more severe MR were more frequently treated with beta-blockers (89.5% vs. 71.4%, *p* = 0.029) and loop diuretics (56.1% vs. 32.7%, *p* = 0.021), reflecting greater symptomatic burden and more advanced HF.

### 3.3. Three-Dimensional Transesophageal Echocardiographic Assessment of Mitral Valve Geometry

**Figure 2:**
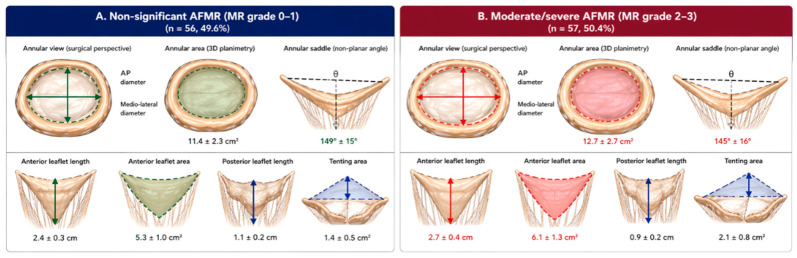
Three-dimensional mitral valve geometry in (**A**) non-significant vs. (**B**) moderate/severe atrial functional mitral regurgitation. Abbreviations: AFMR = atrial functional mitral regurgitation; MR = mitral regurgitation; AP = antero-posterior; 3D = three-dimensional.

Three-dimensional TEE parameters according to MR severity are presented in Table 2 and Figure 2. Image quality was adequate for complete 3D mitral valve analysis in all 113 patients (100% feasibility), reflecting the superior visualization afforded by the transesophageal approach in patients with AF.

#### 3.3.1. Annular Geometry

Patients with moderate or severe AFMR exhibited significantly larger annular dimensions compared with those with non-significant regurgitation. 3D annular area was greater in the moderate/severe group (median 12.7 vs. 11.4 cm^2^, *p* = 0.005), as were annular perimeter (12.9 vs. 12.2 cm, *p* = 0.003) and anteroposterior diameter (3.9 vs. 3.7 cm, *p* = 0.016). Annular height was increased in patients with more severe regurgitation (8.0 vs. 7.0 mm, *p* = 0.007), while the non-planar angle was significantly lower (145° vs. 149°, *p* = 0.027), indicating flattening of the normal saddle-shaped annular configuration. No significant differences were observed in sphericity index, mitral annular excursion, mitral annular maximal velocity, or annulus area fraction, suggesting that static geometry rather than dynamic annular function distinguishes MR severity groups.

#### 3.3.2. Leaflet Geometry

Leaflet dimensions showed striking differences according to MR severity, with the high spatial resolution of 3D TEE enabling precise quantification of these parameters. Patients with moderate or severe regurgitation had significantly larger anterior leaflet area (median 6.1 vs. 5.3 cm^2^, *p* = 0.001) and posterior leaflet area (6.9 vs. 6.2 cm^2^, *p* = 0.021). Most notably, anterior leaflet length was markedly greater in the moderate/severe group (median 2.7 cm [IQR 2.6–3.0] vs. 2.4 cm [IQR 2.2–2.6], *p* = 0.001), representing the most significant difference among all leaflet parameters. In contrast, posterior leaflet length did not differ significantly between groups (1.6 vs. 1.5 cm, *p* = 0.582), suggesting that anterior leaflet elongation, rather than posterior leaflet changes, is the critical adaptive response in AFMR. Posterior leaflet angle was increased in patients with more severe regurgitation (28° vs. 25°, *p* = 0.010), consistent with atriogenic leaflet tethering.

#### 3.3.3. Coaptation and Tethering

Coaptation geometry was significantly altered in patients with more severe regurgitation. Anterior closure line length was greater in the moderate/severe group (3.8 vs. 3.4 cm, *p* = 0.004), as was posterior closure line length (3.8 vs. 3.5 cm, *p* = 0.008). Tethering parameters were consistently more pronounced in patients with moderate or severe MR: tenting height (0.7 vs. 0.6 cm, *p* = 0.011), tenting area (1.9 vs. 1.4 cm^2^, *p* = 0.001), and tenting volume (2.6 vs. 2.0 mL, *p* = 0.012) were all significantly increased, indicating apical displacement of the coaptation zone.

### 3.4. Predictors of Moderate or Severe MR

#### 3.4.1. Univariable Analysis

In univariable logistic regression (Table 3), multiple 3D parameters were significantly associated with moderate or severe MR. Among annular parameters, 3D annular area (OR 1.77 per SD, 95% CI 1.16–2.69, *p* < 0.001), and annular perimeter (OR 1.83 per SD, 95% CI 1.19–2.81, *p* < 0.001) were significant predictors. Among leaflet parameters, anterior leaflet area (OR 2.19 per SD, 95% CI 1.38–3.48, *p* < 0.001), posterior leaflet area (OR 1.55 per SD, 95% CI 1.04–2.32, *p* = 0.032), and posterior leaflet angle (OR 1.74 per SD, 95% CI 1.16–2.60, *p* < 0.001) were associated with MR severity. Tethering parameters including tenting area (OR 1.65 per SD, 95% CI 1.08–2.54, *p* = 0.021) and tenting volume (OR 1.60 per SD, 95% CI 1.07–2.39, *p* = 0.022) were also significant. Among all tested variables, anterior leaflet length demonstrated the strongest association with moderate or severe MR (OR 2.63 per SD increase, 95% CI 1.60–4.30, *p* < 0.001). LA diameter (OR 1.79 per SD, 95% CI 1.10–2.89, *p* = 0.02) and significant TR (OR 3.20, 95% CI 1.38–7.42, *p* < 0.001) were also significant predictors.

#### 3.4.2. Multivariable Analysis

To identify independent predictors of moderate or severe MR while accounting for potential confounding, we constructed a series of exploratory multivariable logistic regression models (Table 4). All models included a common set of baseline covariates: age, sex, LA diameter, and 3D annular area (Model 1, basal model). We then systematically added each leaflet parameter of interest to this basal model to assess its independent contribution beyond annular remodeling. In the basal model (Model 1), 3D annular area remained independently associated with moderate or severe MR (OR 1.77 per SD, 95% CI 1.10–3.05, *p* = 0.025). When anterior leaflet area was added to the basal model (Model 2), it emerged as a strong independent predictor (OR 3.42 per SD, 95% CI 1.48–8.74, *p* = 0.006), while 3D annular area lost its independent significance (OR 0.66, 95% CI 0.27–1.53, *p* = 0.337), suggesting that leaflet area captures the dominant signal previously attributed to annular dilation. Similarly, when anterior leaflet length was added to the basal model (Model 3), it demonstrated robust independent association with moderate or severe MR (OR 3.16 per SD, 95% CI 1.65–6.61, *p* = 0.001), again rendering 3D annular area non-significant (OR 0.84, 95% CI 0.41–1.65, *p* = 0.607). The magnitude and significance of the anterior leaflet length effect were comparable to those of anterior leaflet area, indicating that both anterior leaflet dimensions independently predict MR severity beyond annular geometry. In contrast, posterior leaflet area (Model 4) did not retain independent significance when added to the basal model (OR 0.68, 95% CI 0.24–1.76, *p* = 0.443). Interestingly, posterior leaflet length (Model 5) showed an inverse association with MR severity (OR 0.39, 95% CI 0.15–0.86, *p* = 0.043), while 3D annular area regained significance (OR 2.51, 95% CI 1.37–5.02, *p* = 0.005). This paradoxical finding likely reflects the fact that posterior leaflet length is relatively preserved across MR severity groups (as shown in univariable analysis), and when adjusted for annular size, shorter posterior leaflets may indicate inadequate adaptive growth. Age was not independently associated with MR severity, whereas female sex was independently associated with moderate or severe MR in most models. These findings demonstrate that anterior leaflet dimensions—both area and length—are the strongest independent correlates of moderate or severe AFMR, superseding the predictive value of annular parameters when both are considered simultaneously in multivariable models.

To further evaluate the independent contribution of leaflet geometry parameters, an additional multivariable logistic regression model including anterior leaflet length, anterior leaflet area, and posterior leaflet length simultaneously was performed (Table 5). In this model, female sex (OR 3.20, 95% CI 1.21–9.04, *p* = 0.022), and LA diameter (OR 1.80, 95% CI 1.09–3.14, *p* = 0.027) remained independently associated with moderate/severe AFMR. None of the leaflet-related parameters remained independently associated with MR severity after simultaneous adjustment, suggesting that these measurements convey partially overlapping information regarding leaflet remodeling. Variance inflation factors were below 5 in all multivariable models (maximum 3.76), indicating the absence of problematic multicollinearity. The final multivariable model demonstrated acceptable calibration (Hosmer–Lemeshow *p* = 0.262; Brier score = 0.186).

Given the potential influence of AF chronicity on atrial remodeling, an additional sensitivity analysis was performed including AF duration together with age, sex, LA diameter, 3D annular area, and anterior leaflet length. Although AF duration was associated with AFMR severity in univariable analyses, it did not remain independently associated after adjustment (OR 1.55 per SD, 95% CI 0.98–2.57, *p* = 0.072). In contrast, anterior leaflet length remained a strong independent predictor of moderate/severe AFMR (OR 3.35 per SD, 95% CI 1.72–7.19, *p* < 0.001).

### 3.5. Discriminative Performance of Anterior Leaflet Size

**Figure 3:**
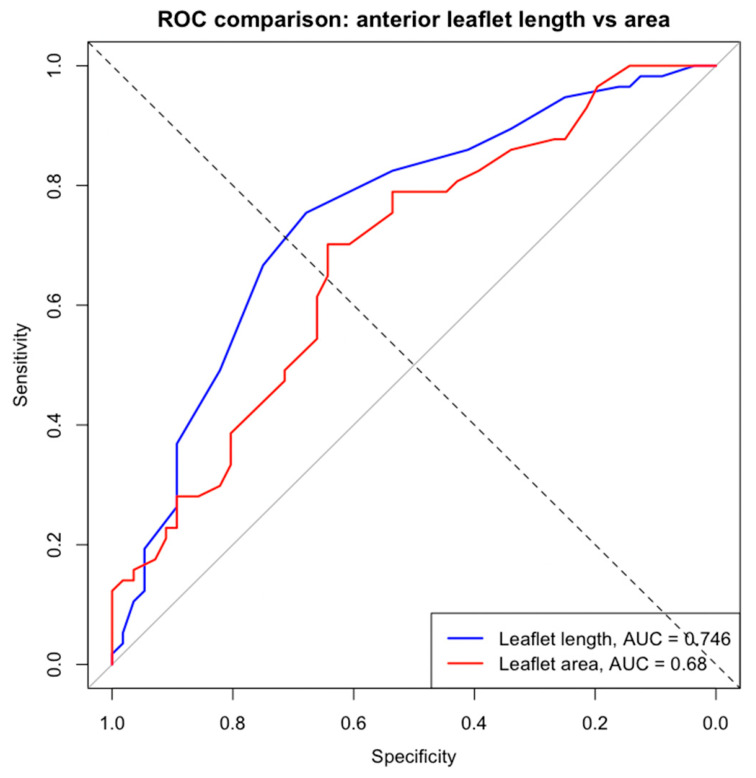
ROC curves comparing anterior leaflet length and area for detecting moderate or severe AFMR. Anterior leaflet length showed higher discrimination (AUC 0.746 vs. 0.680), without a significant difference (*p* = 0.076).

Receiver operating characteristic (ROC) analysis (Figure 3) demonstrated that anterior leaflet length had good discriminative ability for identifying moderate or severe MR, with an area under the curve (AUC) of 0.746 (95% CI 0.654–0.838). The optimal threshold derived from the Youden index was 2.55 cm, yielding a sensitivity of 75.4% and a specificity of 67.9% for detecting moderate or severe AFMR. In comparison, anterior leaflet area showed a lower discriminative performance, with an AUC of 0.680 (95% CI 0.582–0.779). The optimal cut-off value was 5.75 cm^2^, corresponding to a sensitivity of 70.2% and a specificity of 64.3%. However, the difference in AUC between anterior leaflet length and anterior leaflet area did not reach statistical significance (DeLong test: Z = 1.77, *p* = 0.076).

### 3.6. Reproducibility Analysis

Reproducibility analysis demonstrated excellent agreement across all assessed 3D mitral valve parameters. Anterior leaflet length showed high intra- and inter-observer reproducibility (ICC = 0.989 and 0.970, respectively; both *p* < 0.001), with minimal bias and narrow limits of agreement on Bland–Altman analysis. Similarly, excellent inter-observer agreement was observed for 3D mitral annular area (ICC = 0.999, 95% CI 0.997–0.999) and anterior leaflet area (ICC = 0.998, 95% CI 0.996–0.999), both demonstrating very small biases and excellent consistency between measurements. These findings support the robustness and clinical applicability of 3D echocardiographic quantification in AFMR.

## 4. Discussion

The present study provides a comprehensive 3D TEE characterization of the structural parameters associated with AFMR severity in a well-defined cohort of patients with AF and preserved LV function. The principal findings are that anterior leaflet dimensions—both length and area—are the strongest independent predictors of moderate or severe AFMR, outperforming annular area in multivariable analysis. Additionally, posterior leaflet length demonstrated an inverse association with MR severity, suggesting a potential protective role of posterior leaflet elongation. These observations extend prior work on leaflet morphological alterations in functional MR and suggest that simple leaflet measurements may provide clinically actionable information for risk stratification and patient selection [6,7].

### 4.1. Anterior Leaflet Dimensions as Key Parameters Associated with AFMR Severity

Prior 3D echocardiographic studies in AFMR have consistently demonstrated that leaflet-to-annulus area ratio determines regurgitation severity. Kim et al. [6] showed that mitral leaflet area increases in response to annular dilation, but this adaptive remodeling plateaus at higher degrees of dilation, resulting in inadequate leaflet coverage and more severe MR. Kagiyama et al. [7] further demonstrated that patients with significant AFMR have lower leaflet-to-annulus area ratios than those without significant regurgitation. These studies established the paradigm that leaflet area relative to annular size is a critical determinant of coaptation adequacy. Our findings extend this paradigm by demonstrating that both anterior leaflet length and anterior leaflet area are independently associated with moderate or severe AFMR when adjusted for annular dimensions. In multivariable analysis, anterior leaflet length (OR 3.16 per standard deviation increase, 95% CI 1.65–6.61, *p* = 0.001) and anterior leaflet area (OR 3.42 per SD, 95% CI 1.48–8.74, *p* = 0.006) both remained significant predictors after adjustment for age, sex, LA diameter, and 3D annular area. Notably, when either anterior leaflet parameter was added to the basal model, 3D annular area lost its independent significance, suggesting that leaflet dimensions capture the dominant signal previously attributed to annular dilation. The comparable predictive strength of anterior leaflet length and area (AUC 0.746 vs. 0.680, *p* = 0.076 for difference) suggests that both measurements provide clinically useful information. However, anterior leaflet length offers a practical advantage: it is a single linear measurement that can be obtained more readily than area calculations, which require complete leaflet surface reconstruction. The optimal threshold of 2.55 cm for anterior leaflet length (sensitivity 75.4%, specificity 67.9%) may facilitate rapid risk stratification in clinical practice. Yet, an additional multivariable model including all leaflet geometry parameters simultaneously demonstrated that none of the individual leaflet measurements remained independently associated with MR severity. This finding suggests that anterior leaflet length, anterior leaflet area, and posterior leaflet length might provide overlapping information regarding valvular morphology in AFMR. Therefore, leaflet geometry should be interpreted as an integrated structural phenotype rather than as a collection of entirely independent markers—in AFMR, leaflet morphology represents an integrated geometric phenotype in which leaflet elongation, remodeling, coaptation changes, and tethering interact dynamically.

### 4.2. The Paradox of Larger Leaflets in More Severe Regurgitation

An important conceptual consideration is the interpretation of larger anterior leaflet dimensions in patients with more severe MR. At first glance, this finding appears paradoxical: if leaflet enlargement contributed to maintaining coaptation, why would larger leaflets be associated with worse regurgitation? This may reflect the fact that absolute leaflet size does not fully capture leaflet adequacy relative to annular enlargement and tethering. The leaflet-to-annulus mismatch—rather than absolute leaflet size—determines coaptation failure [1,2,3]. This interpretation is supported by our multivariable analysis: when anterior leaflet dimensions are included in models containing 3D annular area, the annular parameter loses significance. This suggests that leaflet size, when considered alongside annular dimensions, captures the critical balance between annular dilation and leaflet morphology. Patients with larger leaflets relative to their annular size maintain coaptation, whereas those with inadequate relative leaflet coverage relative to annular dilation and tethering may develop significant regurgitation. The finding that anterior leaflet length remained independently associated with regurgitation severity even after adjustment for annular dimensions and LA size suggests that leaflet remodeling is not simply a passive response to annular dilation but may represent an active biological process that varies among individuals. Adaptive leaflet growth is a complex process mediated by endothelial–mesenchymal transition, triggered by cellular signaling factors including transforming growth factor-β [2,13]. Some patients may mount a more robust compensatory leaflet growth response, thereby maintaining adequate coaptation despite annular enlargement, whereas others with inadequate leaflet morphology develop more severe regurgitation. An important consideration is that adaptive leaflet growth may not always be beneficial; the appearance of MR despite apparently adequate adaptive leaflet growth may be attributed to “runaway fibrosis,” which causes leaflet stiffening that precludes adequate coaptation [2].

### 4.3. Posterior Leaflet Length: A Protective Factor?

A novel finding of the present study is the inverse association between posterior leaflet length and MR severity in multivariable analysis (OR 0.38 per SD, 95% CI 0.14–0.87, *p* = 0.049). This suggests that greater posterior leaflet elongation may be protective against significant AFMR. Importantly, posterior leaflet length did not differ significantly between groups in univariable analysis (*p* = 0.582), indicating that this protective effect becomes apparent only after adjustment for annular size. This finding has mechanistic plausibility. In the context of atriogenic leaflet tethering, the posterior leaflet is preferentially affected by posterior annular displacement [3,14]. A longer posterior leaflet may better compensate for this tethering effect, maintaining adequate coaptation height despite annular displacement [3]. Conversely, patients with shorter posterior leaflets relative to their annular size may have insufficient tissue to overcome tethering forces, resulting in more severe regurgitation. The differential behavior of anterior and posterior leaflets—with anterior leaflet enlargement marking disease severity and posterior leaflet elongation potentially conferring protection—highlights the complex interplay between leaflet geometry and coaptation mechanics in AFMR.

### 4.4. The Role of 3D TEE

A methodological strength of the present study is the use of 3D TEE for mitral valve assessment. While prior studies of leaflet morphology in AFMR have predominantly used 3D transthoracic echocardiography, the transesophageal approach offers superior spatial resolution for precise leaflet measurements [6,7]. This is particularly relevant for accurate quantification of leaflet length, which requires clear delineation of the annular hinge point and leaflet free edge—structures that may be suboptimally visualized with transthoracic imaging, especially in patients with AF and irregular R-R intervals. The excellent reproducibility of anterior leaflet length measurements in our study (ICC 0.989 intra-observer, 0.970 inter-observer) supports the feasibility of this parameter for clinical application. Similarly high reproducibility was observed for anterior leaflet area (ICC 0.998) and 3D annular area (ICC 0.999). The 100% feasibility rate for complete 3D analysis further underscores the advantages of the transesophageal approach in this patient population.

### 4.5. Annular Remodeling in AFMR

Mitral annular dilation has long been recognized as the primary mechanism underlying AFMR [2]. The present study confirms and extends these observations by demonstrating that patients with moderate or severe AFMR have significantly larger 3D annular area (12.7 vs. 11.4 cm^2^, *p* = 0.005), perimeter (12.9 vs. 12.2 cm, *p* = 0.003), and anteroposterior diameter (3.9 vs. 3.7 cm, *p* = 0.016) compared with those with milder regurgitation. The pathophysiological cascade linking AF to annular dilation involves loss of atrial contractility, chronic elevation of LA pressure, progressive atrial enlargement, and subsequent annular expansion [1,2,15]. However, the present analysis reveals that annular remodeling in AFMR extends beyond simple planar enlargement to include complex 3D geometric distortion. Patients with more severe regurgitation demonstrated flattening of the normal saddle-shaped annular configuration, as evidenced by a lower non-planar angle (145° vs. 149°, *p* = 0.027) despite paradoxically increased annular height (8.0 vs. 7.0 mm, *p* = 0.007). The loss of the saddle shape may have important functional consequences beyond its effect on annular area, as the normal saddle-shaped configuration is thought to reduce leaflet stress and optimize coaptation geometry [15]. Interestingly, dynamic annular parameters, including annular excursion, maximal velocity, and area fraction, did not differ significantly between groups. This suggests that the primary determinant of regurgitation severity in AFMR is structural geometry rather than dynamic annular function, possibly reflecting the fact that all patients in this cohort had AF and therefore had already lost atriogenic annular contraction [1,2]. Our findings also provide indirect support for the role of AF chronicity in the development of AFMR. Patients with moderate or severe AFMR had a significantly longer duration of AF compared with those with non-significant MR. However, AF duration did not remain independently associated with AFMR severity after adjustment for LA size and mitral valve geometric parameters. This finding suggests that the effect of AF chronicity is largely mediated through structural remodeling, whereas leaflet geometry retains independent relevance for AFMR severity.

### 4.6. Comparison with Ventricular Functional MR

Our findings parallel observations in ventricular functional MR. Studies in patients with dilated cardiomyopathy [16] have demonstrated that inadequate anterior leaflet elongation contributes to functional MR severity, with the anterior leaflet showing greater adaptive capacity than the posterior leaflet [13]. The present study extends this observation to the atrial functional phenotype, suggesting that anterior leaflet morphology may be a universal determinant of functional MR severity, regardless of whether the primary pathology is atrial or ventricular. Uno et al. [14] compared mitral valve geometry between atrial and ventricular functional MR using 3D TEE and found distinct patterns of leaflet and annular remodeling. Our findings complement this work by demonstrating that within the AFMR population, anterior leaflet dimensions are the strongest independent predictors of severity. This finding has important implications for understanding AFMR pathophysiology. Silbiger’s atriogenic leaflet tethering [3] model emphasizes posterior leaflet involvement, proposing that posterior annular displacement and inward bending of the basal posterior left ventricle cause preferential tethering of the posterior leaflet. However, our data suggest that the anterior leaflet may be equally or more important in determining coaptation adequacy. One possible explanation is that while posterior leaflet tethering initiates malcoaptation, the anterior leaflet’s capacity to compensate by elongating determines the ultimate severity of regurgitation.

### 4.7. Tethering and Coaptation Abnormalities

The present study demonstrates that patients with moderate or severe AFMR exhibit significantly greater tethering parameters, including tenting height (0.7 vs. 0.6 cm, *p* = 0.011), tenting area (1.9 vs. 1.4 cm^2^, *p* = 0.001), and tenting volume (2.6 vs. 2.0 mL, *p* = 0.012), compared with those with milder regurgitation. The concept of atriogenic leaflet tethering has been proposed as a key mechanism in AFMR pathophysiology [2,3]. As the LA enlarges, the posterior mitral annulus becomes displaced onto the crest and eventually onto the epicardial surface of the posterobasal wall of the LV. This displacement causes the posterior mitral leaflet to be tethered out of the annular plane, increasing the posterior leaflet angle and reducing the effective height of the residual portion of the posterior leaflet [3]. The present study confirms this mechanism, demonstrating significantly increased posterior leaflet angle in patients with more severe regurgitation (28° vs. 25°, *p* = 0.010).

### 4.8. The Broader Remodeling Phenotype

Beyond leaflet geometry, our study confirms that AFMR severity is associated with a coherent pattern of structural remodeling involving the annulus, leaflets, and subvalvular apparatus. Patients with moderate or severe AFMR exhibited larger annular dimensions, reduced non-planar angle indicating annular flattening, increased leaflet areas, and more pronounced tethering. This integrated phenotype supports the concept that AFMR progression reflects a complex remodeling process rather than isolated annular dilation [1,15]. The frequent coexistence of significant TR (43.9% in the moderate/severe AFMR group vs. 19.6% in the non-significant group, *p* = 0.010) further supports the notion of a broader atrial cardiomyopathy affecting both atrioventricular valves [17]. Atrial functional TR has recently been recognized as a distinct pathophysiological entity driven by right atrial remodeling and annular dilation, paralleling mechanisms observed in AFMR [18]. This biatrial remodeling phenotype has implications for surgical and transcatheter planning, as concomitant tricuspid intervention may be warranted in patients with AFMR undergoing mitral valve procedures.

### 4.9. Clinical Implications: Simplicity and Practicality

A key strength of our findings is the potential for clinical translation. Prior studies have proposed complex indices such as leaflet-to-closure area ratio or leaflet-to-annulus area ratio to predict AFMR severity [6,7]. While mechanistically informative, these ratios require dedicated 3D reconstruction and offline analysis, limiting their applicability in routine clinical practice. In contrast, anterior leaflet length is a single linear measurement that can be obtained from standard 3D mitral valve datasets using widely available software. Our analysis identified a threshold of 2.55 cm, above which moderate or severe AFMR was more likely (AUC 0.746, 95% CI 0.654–0.838; sensitivity 75.4%, specificity 67.9%). From a clinical standpoint, integrating this 2.55 cm threshold into routine evaluation can refine patient management across three major axes: optimizing patient selection for interventions, enhancing longitudinal surveillance to catch adaptive mismatches early, and defining a tight therapeutic window to guide the timing of rhythm control versus valvular intervention. Anterior leaflet area, with a threshold of 5.75 cm^2^ (AUC 0.680), provides an alternative metric. If validated in external cohorts, these simple measurements could facilitate rapid risk stratification and patient selection for intervention. This has particular relevance for transcatheter edge-to-edge repair (TEER), which has emerged as an important therapeutic option for AFMR [19,20]. Recent registry data from the OCEAN-Mitral and REVEAL-AFMR registries suggest that TEER is associated with improved outcomes in AFMR compared with medical therapy alone, particularly when residual MR is reduced to mild or less [20,21,22]. However, patient selection remains challenging, and predictors of procedural success and durability are needed. Studies have demonstrated that insufficient leaflet remodeling relative to annular dilation predicts residual MR after MitraClip implantation [22]. Our finding that anterior leaflet dimensions have the strongest independent association with AFMR severity suggests that these parameters may also predict response to TEER—a hypothesis that warrants prospective investigation. Furthermore, our results may inform device development. Current annuloplasty-based approaches target annular reduction but do not address leaflet insufficiency [23]. Patients with inadequate anterior leaflet morphology may benefit from alternative strategies, such as leaflet augmentation or combined annuloplasty with edge-to-edge repair, to achieve durable MR reduction.

### 4.10. Relationship with Heart Failure with Preserved Ejection Fraction

The present study population is representative of the typical AFMR phenotype, with preserved LV ejection fraction (median 51%) and a high prevalence of HF (80.5%). The relationship between AFMR and HFpEF is bidirectional and complex [5]. Diastolic dysfunction and increased LA pressures, due to neurohormonal imbalances including depletion of atrial natriuretic peptide and activation of the renin–angiotensin–aldosterone system, account for a major role in atrial remodeling, which facilitates initiation and maintenance of both AFMR and AF [2,5]. The present study’s finding that patients with moderate or severe AFMR were more frequently treated with loop diuretics (56.1% vs. 32.7%, *p* = 0.021) suggests greater hemodynamic stress and more advanced HF. The natural history of AFMR appears complex; Naser et al. [5] found that regression of MR was more common than progression over a median 2.2-year follow-up, though independent risk factors for mortality included older age, concentric LV geometry, and higher LV filling pressures rather than MR severity per se.

### 4.11. Study Limitations

Several limitations should be acknowledged. First, this was a single-center observational study, and the findings require external validation in independent cohorts. Second, the cross-sectional design precludes assessment of temporal changes in mitral valve geometry and their relationship to disease progression. Third, the sample size (*n* = 113) limited the number of variables that could be included in multivariable modeling; the exploratory nature of the multivariable models should be emphasized. Fourth, the proposed anterior leaflet length threshold of 2.55 cm and anterior leaflet area threshold of 5.75 cm^2^ are exploratory and derived from the same cohort used for model development; prospective validation is essential before clinical application. Fifth, we did not assess clinical outcomes such as HF hospitalization or mortality; whether anterior leaflet dimensions predict prognosis or response to intervention remains to be determined. Sixth, all measurements were performed during AF, and the influence of rhythm on leaflet geometry could not be assessed. Finally, while 3D TEE provides superior image quality, and enabled complete mitral valve quantification in all patients included in the present study, the high feasibility observed may partly reflect the use of dedicated TEE imaging in a high-volume tertiary center and may not be generalizable to all clinical settings. Moreover, 3D TEE is more invasive than transthoracic imaging, and whether similar findings would be obtained with high-quality 3D TTE requires further investigation.

## 5. Conclusions

In patients with AFMR, anterior leaflet dimensions—both length and area—assessed by 3D TEE are the strongest independent predictors of moderate or severe regurgitation, outperforming annular area when both are considered in multivariable models. Posterior leaflet length demonstrated an inverse association with MR severity, suggesting a potential protective role of posterior leaflet elongation. These measurements may offer practical tools for risk stratification and patient selection. The finding that leaflet dimensions, rather than annular size alone, determine coaptation adequacy extends prior observations in ventricular functional MR to the atrial phenotype and underscores the importance of comprehensive leaflet assessment in AFMR evaluation. Prospective studies are needed to validate the proposed thresholds and to determine whether anterior leaflet dimensions predict clinical outcomes and response to transcatheter or surgical intervention.

## Figures and Tables

**Table 1:** Baseline Clinical and Echocardiographic Characteristics.

Variable	Overall (*n* = 113)	MR < Moderate (*n* = 56)	MR ≥ Moderate (*n* = 57)	*p*-Value
Demographics				
Age, years	68 [63–73]	67 [61–70]	70 [65–74]	0.044
Female sex	43 (38.1%)	17 (30.4%)	26 (45.6%)	0.140
CHA2DS2-VASc score	3 [2–4]	3 [2–3]	3 [2–4]	0.180
AF duration, months	2 [1–6]	2 [1–4]	3 [2–8]	0.002
Long-standing persistent AF	18 (15.9%)	6 (10.7%)	12 (21.1%)	0.21
Comorbidities				
Hypertension	100 (89.3%)	48 (87.3%)	52 (91.2%)	0.711
Diabetes mellitus	39 (34.5%)	17 (30.4%)	22 (38.6%)	0.470
Dyslipidemia	96 (85.0%)	45 (80.4%)	51 (89.5%)	0.275
Coronary artery disease	13 (11.5%)	5 (8.9%)	8 (14.0%)	0.578
Heart failure	91 (80.5%)	41 (73.2%)	50 (87.7%)	0.087
Chronic kidney disease	39 (34.5%)	17 (30.4%)	22 (38.6%)	0.470
Medications				
Beta-blocker	91 (80.5%)	40 (71.4%)	51 (89.5%)	0.029
RAAS inhibitor	88 (77.9%)	42 (75.0%)	46 (80.7%)	0.615
Loop diuretic	50 (44.6%)	18 (32.7%)	32 (56.1%)	0.021
Oral anticoagulation	107 (94.7%)	55 (98.2%)	52 (91.2%)	0.206
SGLT2 inhibitor	21 (18.6%)	7 (12.5%)	14 (24.6%)	0.160
Laboratory				
Creatinine, mg/dL	1.0 [0.8–1.2]	1.0 [0.8–1.1]	1.0 [0.8–1.3]	0.522
eGFR, mL/min/1.73 m^2^	69 [55–81]	69 [57–83]	68 [54–77]	0.443
Hemoglobin, g/dL	14.3 [13.2–15.3]	14.7 [13.9–15.6]	13.6 [12.8–14.8]	0.003
Standard Echocardiography				
EROA, mm^2^	10 [10–30]	10 [10–10]	30 [20–40]	<0.001
Regurgitant volume, mL	20 [10–38]	10 [8.8–12]	38 [32–48]	<0.001
Vena contracta width	3 [2–5]	2 [1–2]	5 [4–7]	<0.001
LVEF, %	51 [50–55]	53 [50–55]	50 [45–55]	0.198
Left atrial diameter, mm	45 [41–48]	44 [40–47]	46 [42–50]	0.005
Right atrial diameter, mm	40 [37–43]	39 [36–41]	41 [38–45]	0.041
sPAP, mmHg	33 [25–41]	30 [27–36]	35 [25–45]	0.381
TR ≥ moderate	36 (31.9%)	11 (19.6%)	25 (43.9%)	0.010

Continuous variables are reported as median [interquartile range]; categorical variables as *n* (%). AF = atrial fibrillation; eGFR = estimated glomerular filtration rate; LVEF = left ventricular ejection fraction; MR = mitral regurgitation; RAAS = renin–angiotensin–aldosterone system; SGLT2 = sodium-glucose cotransporter-2; sPAP = systolic pulmonary artery pressure; TR = tricuspid regurgitation.

**Table 2:** Three-dimensional transesophageal echocardiographic parameters.

Parameter	Overall (*n* = 113)	MR Moderate (*n* = 56)	MR ≥ Moderate (*n* = 57)	*p*-Value
Annular Geometry				
3D annular area, cm^2^	12.1 [10.4–13.6]	11.4 [10.0–12.8]	12.7 [11.3–13.7]	0.005
2D annular area, cm^2^	11.2 [9.8–12.8]	10.7 [9.4–12.2]	11.6 [10.3–12.9]	0.028
Annular perimeter, cm	12.5 [11.6–13.2]	12.2 [11.4–12.8]	12.9 [12.1–13.3]	0.003
Anteroposterior diameter, cm	3.7 [3.4–4.1]	3.7 [3.3–4.0]	3.9 [3.6–4.1]	0.016
PM-AL diameter, cm	3.7 [3.5–4.0]	3.7 [3.4–3.9]	3.8 [3.6–4.0]	0.099
Commissural diameter, cm	3.6 [3.4–3.8]	3.6 [3.3–3.8]	3.7 [3.5–3.9]	0.198
Inter-trigonal distance, cm	2.7 [2.4–2.8]	2.7 [2.4–2.8]	2.7 [2.4–2.9]	0.326
Sphericity index	1.0 [0.9–1.1]	1.0 [0.9–1.1]	1.0 [0.9–1.1]	0.525
Annular height, mm	7.6 [5.6–8.9]	7.0 [4.5–8.3]	8.0 [6.3–9.4]	0.007
Non-planar angle, °	146 [138–157]	149 [141–162]	145 [135–152]	0.027
Mitral annular excursion, mm	3.6 [2.4–5.2]	3.5 [2.4–4.9]	3.7 [2.4–5.5]	0.613
Mitral annular max velocity, cm/s	23.7 [18.1–31.4]	24.4 [17.5–31.5]	23.4 [19.5–31.3]	0.683
Annulus area fraction, %	0.7 [0.3–1.3]	0.7 [0.3–1.5]	0.6 [0.3–1.1]	0.289
Leaflet Geometry				
Anterior leaflet area, cm^2^	5.9 [5.0–6.6]	5.3 [4.8–6.3]	6.1 [5.6–7.1]	0.001
Posterior leaflet area, cm^2^	6.7 [5.6–7.8]	6.2 [5.4–7.3]	6.9 [5.9–8.1]	0.021
Anterior leaflet length, cm	2.6 [2.3–2.8]	2.4 [2.2–2.6]	2.7 [2.6–3.0]	0.001
Posterior leaflet length, cm	1.6 [1.3–1.8]	1.5 [1.3–1.7]	1.6 [1.3–1.9]	0.582
Anterior leaflet angle, °	15 [12–19]	14 [11–17]	16 [12–19]	0.115
Posterior leaflet angle, °	26 [19–33]	25 [16–30]	28 [23–33]	0.010
Coaptation and Tethering				
Anterior closure line length, cm	3.6 [3.3–4.1]	3.4 [3.1–3.9]	3.8 [3.4–4.2]	0.004
Posterior closure line length, cm	3.6 [3.3–4.1]	3.5 [3.2–3.9]	3.8 [3.4–4.3]	0.008
Tenting height, cm	0.6 [0.5–0.8]	0.6 [0.4–0.7]	0.7 [0.6–0.9]	0.011
Tenting area, cm^2^	1.6 [1.2–2.2]	1.4 [1.1–1.8]	1.9 [1.4–2.5]	0.001
Tenting volume, mL	2.2 [1.5–3.4]	2.0 [1.2–2.7]	2.6 [1.8–3.7]	0.012
Tenting volume fraction, %	23.3 [10.9–36.0]	24.1 [11.7–36.2]	22.6 [10.9–34.6]	0.995

Continuous variables are reported as median [interquartile range]. 3D = three-dimensional; MR = mitral regurgitation; PM-AL = posteromedial-anterolateral.

**Table 3:** Univariable logistic regression analysis for moderate or severe MR.

Variable	OR (95% CI)	*p*-Value
Clinical Variables		
Age, per 1 SD	1.35 (0.92–2.01)	0.13
Female sex	1.92 (0.89–4.16)	0.10
Left atrial diameter, per 1 SD	1.79 (1.10–2.89)	0.02
TR ≥ moderate	3.20 (1.38–7.42)	<0.001
Annular Parameters		
3D annular area, per 1 SD	1.77 (1.16–2.69)	<0.001
Annular perimeter, per 1 SD	1.83 (1.19–2.81)	<0.001
Anteroposterior diameter, per 1 SD	1.64 (1.10–2.45)	0.02
Non-planar angle, per 1 SD	0.80 (0.52–1.21)	0.29
Leaflet Parameters		
Anterior leaflet area, per 1 SD	2.19 (1.38–3.48)	<0.001
Posterior leaflet area, per 1 SD	1.55 (1.04–2.32)	0.03
Anterior leaflet length, per 1 SD	2.63 (1.60–4.30)	<0.001
Posterior leaflet length, per 1 SD	0.86 (0.51–1.27)	0.48
Anterior leaflet angle, per 1 SD	1.34 (0.92–2)	0.14
Posterior leaflet angle, per 1 SD	1.74 (1.16–2.60)	<0.001
Tethering Parameters		
Anterior closure line length, per 1 SD	1.88 (1.22–2.90)	<0.001
Posterior closure line length, per 1 SD	1.73 (1.12–2.68)	0.01
Tenting height, per 1 SD	1.46 (0.98–2.18)	0.06
Tenting area, per 1 SD	1.65 (1.08–2.54)	0.02
Tenting volume, per 1 SD	1.60 (1.07–2.39)	0.02

Continuous predictors were standardized per 1 standard deviation increase. CI = confidence interval; OR = odds ratio; SD = standard deviation.

**Table 4:** Multivariable logistic regression analyses for moderate or severe MR.

Variable	OR (95% CI)	*p*-Value
Model 1 (Basal Model)		
Age (per 1 SD)	1.20 (0.79–1.84)	0.395
Female sex	2.69 (1.13–6.69)	0.028
Left atrial diameter (per 1 SD)	1.57 (0.96–2.71)	0.085
3D annular area (per 1 SD)	1.77 (1.10–3.05)	0.025
**Model 2 (basal model + anterior leaflet area)**		
Age (per 1 SD)	1.24 (0.82–1.93)	0.314
Female sex	2.92 (1.19–7.57)	0.022
Left atrial diameter (per 1 SD)	1.71 (1.04–2.98)	0.045
3D annular area (per 1 SD)	0.66 (0.27–1.53)	0.337
Anterior leaflet area (per 1 SD)	3.42 (1.48–8.74)	0.006
**Model 3 (basal model + anterior leaflet length)**		
Age (per 1 SD)	1.22 (0.79–1.92)	0.374
Female sex	2.56 (1.02–6.74)	0.050
Left atrial diameter (per 1 SD)	1.73 (1.07–2.99)	0.034
3D annular area (per 1 SD)	0.84 (0.41–1.65)	0.607
Anterior leaflet length (per 1 SD)	3.16 (1.65–6.61)	0.001
**Model 4 (basal model + posterior leaflet area)**		
Age (per 1 SD)	1.18 (0.78–1.81)	0.442
Female sex	2.64 (1.11–6.58)	0.032
Left atrial diameter (per 1 SD)	1.62 (0.99–2.81)	0.069
3D annular area (per 1 SD)	2.47 (0.95–7.06)	0.074
Posterior leaflet area (per 1 SD)	0.68 (0.24–1.76)	0.443
**Model 5 (basal model + posterior leaflet length)**		
Age (per 1 SD)	1.18 (0.77–1.85)	0.453
Female sex	3.67 (1.45–10.01)	0.008
Left atrial diameter (per 1 SD)	1.60 (0.98–2.79)	0.077
3D annular area (per 1 SD)	2.51 (1.37–5.02)	0.005
Posterior leaflet length (per 1 SD)	0.39 (0.15–0.86)	0.043

Continuous predictors were standardized per 1 standard deviation increase. CI = confidence interval; OR = odds ratio; SD = standard deviation.

**Table 5:** Multivariable logistic regression model including all leaflet geometry parameters for moderate or severe MR.

Variable	OR (95% CI)	*p*-Value
Age (per 1 SD)	1.26 (0.81–1.99)	0.311
Female sex	3.2 (1.21–9.04)	0.022
Left atrial diameter (per 1 SD)	1.80 (1.09–3.14)	0.027
3D annular area (per 1 SD)	0.64 (0.24–1.80)	0.363
Anterior leaflet length (per 1 SD)	2.13 (0.98–4.90)	0.063
Anterior leaflet area (per 1 SD)	2.22 (0.85–6.28)	0.115
Posterior leaflet length (per 1 SD)	0.74 (0.31–1.20)	0.315

Continuous predictors were standardized per 1 standard deviation increase. CI = confidence interval; OR = odds ratio; SD = standard deviation.

## Data Availability

The raw data supporting the conclusions of this article will be made available by the authors upon reasonable request and following approval by the University of Medicine and Pharmacy of Craiova, Romania. The data presented in this study are not publicly available due to privacy and ethical restrictions.

## Abbreviations

The following abbreviations are used in this manuscript:

AF	Atrial fibrillation
AFMR	Atrial functional mitral regurgitation
AUC	Area under the curve
CI	Confidence interval
CKD-EPI	Chronic kidney disease epidemiology collaboration
ESC	European society of cardiology
EACTS	European association for cardio-thoracic surgery
EACVI	European association of cardiovascular imaging
EHRA	European heart rhythm association
HF	Heart failure
ICC	Intraclass correlation coefficient
IQR	Interquartile range
LA	Left atrium
LVEF	Left ventricular ejection fraction
MR	Mitral regurgitation
OR	Odds ratio
PM-AL	Posteromedial-anterolateral
RAAS	Renin–angiotensin–aldosterone system
ROC	Receiver operating characteristics
SD	Standard deviation
SGLT2	Sodium-glucose cotransporter-2
sPAP	Systolic pulmonary artery pressure
TEE	Transesophageal echocardiography
TEER	Transcatheter edge-to-edge repair
TR	Tricuspid regurgitation
TTE	Transthoracic echocardiography
VFMR	Ventricular functional mitral regurgitation
3D	Three-dimensional
2D	Two-dimensional

## References

[1] Deferm S., Bertrand P.B., Verbrugge F.H., Verhaert D., Rega F., Thomas J.D., Vandervoort P.M. (2019). Atrial Functional Mitral Regurgitation: JACC Review Topic of the Week. J. Am. Coll. Cardiol..

[2] Farhan S., Silbiger J.J., Halperin J.L., Zhang L., Dukkipati S.R., Vogel B., Kini A., Sharma S., Lerakis S. (2022). Pathophysiology, Echocardiographic Diagnosis, and Treatment of Atrial Functional Mitral Regurgitation: JACC State-of-the-Art Review. J. Am. Coll. Cardiol..

[3] Silbiger J.J. (2019). Mechanistic Insights into Atrial Functional Mitral Regurgitation: Far More Complicated than Just Left Atrial Remodeling. Echocardiography.

[4] Murata A., Kaneko T., Amano M., Sato Y., Ohno Y., Obokata M., Sato K., Okada T., Sakamoto A., Hirose N. (2025). Qualitative and Quantitative Assessment of Atrial Functional Mitral Regurgitation: Analysis from the REVEAL-AFMR Registry. Eur. Heart J. Cardiovasc. Imaging.

[5] Naser J.A., Alexandrino F.B., Harada T., Michelena H.I., Borlaug B.A., Eleid M.F., Lin G., Scott C., Kennedy A.M., Pellikka P.A. (2024). The Natural History of Atrial Functional Mitral Regurgitation. JACC J. Am. Coll. Cardiol..

[6] Kim D.-H., Heo R., Handschumacher M.D., Lee S., Choi Y.-S., Kim K.-R., Shin Y., Park H.-K., Bischoff J., Aikawa E. (2019). Mitral Valve Adaptation to Isolated Annular Dilation. JACC Cardiovasc. Imaging.

[7] Kagiyama N., Mondillo S., Yoshida K., Mandoli G.E., Cameli M. (2020). Subtypes of Atrial Functional Mitral Regurgitation: Imaging Insights into Their Mechanisms and Therapeutic Implications. JACC Cardiovasc. Imaging.

[8] Caravita S., Figliozzi S., Florescu D.R., Volpato V., Oliverio G., Tomaselli M., Torlasco C., Muscogiuri G., Cernigliaro F., Parati G. (2021). Recent Advances in Multimodality Imaging of the Tricuspid Valve. Expert Rev. Med. Devices.

[9] Gheorghe L.L., Mobasseri S., Agricola E., Wang D.D., Milla F., Swaans M., Pandis D., Adams D.H., Yadav P., Sievert H. (2021). Imaging for Native Mitral Valve Surgical and Transcatheter Interventions. JACC Cardiovasc. Imaging.

[10] Lancellotti P., Pibarot P., Chambers J., La Canna G., Pepi M., Dulgheru R., Dweck M., Delgado V., Garbi M., Vannan M.A. (2022). Multi-Modality Imaging Assessment of Native Valvular Regurgitation: An EACVI and ESC Council of Valvular Heart Disease Position Paper. Eur. Heart J. Cardiovasc. Imaging.

[11] McDonagh T.A., Metra M., Adamo M., Baumbach A., Böhm M., Burri H., Čelutkiene J., Chioncel O., Cleland J.G.F., Coats A.J.S. (2021). 2021 ESC Guidelines for the Diagnosis and Treatment of Acute and Chronic Heart Failure. Eur. Heart J..

[12] Volpato V., Badano L.P., Figliozzi S., Florescu D.R., Parati G., Muraru D. (2021). Multimodality Cardiac Imaging and New Display Options to Broaden Our Understanding of the Tricuspid Valve. Curr. Opin. Cardiol..

[13] Chaput M., Handschumacher M.D., Tournoux F., Hua L., Guerrero J.L., Vlahakes G.J., Levine R.A. (2008). Mitral Leaflet Adaptation to Ventricular Remodeling. Circulation.

[14] Uno G., Omori T., Shimada S., Rader F., Siegel R.J., Shiota T. (2021). Differences in Mitral Valve Geometry between Atrial and Ventricular Functional Mitral Regurgitation in Patients with Atrial Fibrillation: A 3D Transoesophageal Echocardiography Study. Eur. Heart J. Cardiovasc. Imaging.

[15] Zoghbi W.A., Levine R.A., Flachskampf F., Grayburn P., Gillam L., Leipsic J., Thomas J.D., Kwong R.Y., Vandervoort P., Chandrashekhar Y. (2022). Atrial Functional Mitral Regurgitation. JACC Cardiovasc. Imaging.

[16] Kang Y., Chen C., Chen X., Sun X., Wang F., Li L., Zhang Q., Liang Y. (2016). Pattern of Mitral Leaflet Elongation and Its Association with Functional Mitral Regurgitation in Nonischemic Dilated Cardiomyopathy. Am. J. Cardiol..

[17] Utsunomiya H., Itabashi Y., Mihara H., Berdejo J., Kobayashi S., Siegel R.J., Shiota T. (2017). Functional Tricuspid Regurgitation Caused by Chronic Atrial Fibrillation: A Real-Time 3-Dimensional Transesophageal Echocardiography Study. Circ. Cardiovasc. Imaging.

[18] Florescu D.R., Muraru D., Volpato V., Gavazzoni M., Caravita S., Tomaselli M., Ciampi P., Florescu C., Bălșeanu T.A., Parati G. (2022). Atrial Functional Tricuspid Regurgitation as a Distinct Pathophysiological and Clinical Entity: No Idiopathic Tricuspid Regurgitation Anymore. J. Clin. Med..

[19] Vahanian A., Beyersdorf F., Praz F., Milojevic M., Baldus S., Bauersachs J., Capodanno D., Conradi L., De Bonis M., De Paulis R. (2022). 2021 ESC/EACTS Guidelines for the Management of Valvular Heart Disease. Eur. Heart J..

[20] Obadia J.-F., Messika-Zeitoun D., Leurent G., Iung B., Bonnet G., Piriou N., Lefèvre T., Piot C., Rouleau F., Carrié D. (2018). Percutaneous Repair or Medical Treatment for Secondary Mitral Regurgitation. N. Engl. J. Med..

[21] Stone G.W., Lindenfeld J., Abraham W.T., Kar S., Lim D.S., Mishell J.M., Whisenant B., Grayburn P.A., Rinaldi M., Kapadia S.R. (2018). Transcatheter Mitral-Valve Repair in Patients with Heart Failure. N. Engl. J. Med..

[22] Kaneko T., Kagiyama N., Okazaki S., Amano M., Sato Y., Ohno Y., Obokata M., Sato K., Morita K., Kubo S. (2026). Transcatheter Edge-to-Edge Repair vs Medical Therapy in Atrial Functional Mitral Regurgitation: A Propensity Score-Based Comparison from the OCEAN-Mitral and REVEAL-AFMR Registries. Eur. Heart J..

[23] Sakaguchi T., Totsugawa T., Orihashi K., Kihara K., Tamura K., Hiraoka A., Chikazawa G., Yoshitaka H. (2019). Mitral Annuloplasty for Atrial Functional Mitral Regurgitation in Patients with Chronic Atrial Fibrillation. J. Card. Surg..

